# Efficacy of Warm Acupuncture Therapy Combined with Kegel Exercise on Postpartum Pelvic Floor Dysfunction in Women

**DOI:** 10.1007/s00192-023-05698-9

**Published:** 2024-01-18

**Authors:** Jinling Dai, Zhu Jin, Xiaojin Zhang, Feng Lian, Jie Tu

**Affiliations:** 1 Shanghai University of Traditional Chinese Medicine, City Seven Clinical Medical College, Shanghai, 201203 China; 2 Department of Acupuncture, Seventh People’s Hospital of Shanghai University of Traditional Chinese Medicine, Shanghai, 200137 China; 3 Department of Ultrasound, Seventh People’s Hospital of Shanghai University of Traditional Chinese Medicine, Shanghai, 200137 China

**Keywords:** Postpartum pelvic floor dysfunction, Warm acupuncture, Zhibian (BL54) acupoint, Kegel exercise

## Abstract

**Introduction and hypothesis:**

The objective was to observe the clinical efficacy of warm acupuncture combined with Kegel exercise in treating postpartum pelvic floor dysfunction in women.

**Methods:**

A total of 70 primiparous women with postpartum pelvic floor muscle (PFM) injury were randomly divided into a combination group (*n* = 35, receiving warm acupuncture at Zhibian (BL54) acupoint and Kegel exercise) and a sham control group (*n* = 35, receiving sham warm acupuncture and Kegel exercise). Both groups were treated three times a week for 4 consecutive weeks. The recovery of PFM strength and changes in Urethral Rotation Angle (URA), Bladder Neck Descent (BND), and Retrovesical Angle (RVA) in pelvic floor ultrasound reports, the scores of pelvic floor dysfunction-related questionnaires, and the efficacy of urinary incontinence treatment of the two groups were compared before and after treatment.

**Results:**

After treatment, the recovery rates of type I and II PFM strength, pelvic floor ultrasound parameters, pelvic floor dysfunction-related scale scores, and urinary incontinence treatment efficacy in the combination group were significantly better than those in the sham control group (*p* < 0.05).

**Conclusion:**

Warm acupuncture combined with Kegel exercise can significantly improve PFM strength and promote the recovery of postpartum pelvic floor function in women.

## Introduction

Postpartum pelvic floor dysfunction (PPFD) is a common condition among women after childbirth. It is estimated that up to 99.75% of women who have vaginal delivery experience abnormal pelvic floor muscle (PFM) function within 6–8 weeks postpartum [1], with one third of them experiencing urinary incontinence and up to 10% experiencing fecal incontinence [2]. PPFD can have a significant impact on a woman’s quality of life, affecting her physical, emotional, and social well-being. Therefore, effective treatment and management of PPFD are essential. Research shows that after vaginal delivery, the incidence of pelvic floor dysfunction is related to PFM strength. Improving PFM strength can reduce the incidence of pelvic floor dysfunction within 6–12 months after delivery [3, 4]. Kegel exercises are currently the first-line treatment for pelvic floor dysfunction [5, 6]. Although simple PFM training can help women to improve PFM strength after delivery, it does not affect postpartum stress urinary incontinence (SUI) symptoms in women [7]. Therefore, supplemental treatments that can promote postpartum pelvic floor function recovery are needed.

Acupuncture is a traditional Chinese medicine therapy that has been used for thousands of years to treat various conditions, including musculoskeletal and neurological disorders [8–10]. The warming acupuncture technique is a type of acupuncture method in which the needle is heated by placing a burning moxa stick on the handle during the needling process [11]. The combination of mechanical stimulation from needling and thermal stimulation from moxibustion creates a composite stimulus that concentrates heat and effectively penetrates deep into the body. The acupoint Zhibian (BL54) is situated on the Bladder Meridian of Foot-Taiyang, with nerve distribution in the genital area below it. Acupuncture stimulation of these nerves has been shown to enhance PFM strength and urinary control capacity [12].

Chinese women return to the hospital for postpartum examination on the 42nd day after giving birth. The obstetricians recommend that pregnant women undergo PFM strength evaluation, especially those who give birth naturally, to understand the recovery of pelvic floor function in women after giving birth. Therefore, we selected parturients whose PFM strength evaluation showed that both class I and class II PFM strength were < level 3 within 42 days after delivery [13, 14], and who used the treatment method of warm acupuncture combined with Kegel exercise to promote pelvic floor rehabilitation, in order to provide a reference for postpartum rehabilitation of parturients.

## Materials and Methods

### General Data

The present study recruited postpartum women who had experienced PFM injury and sought outpatient follow-up at the Seventh People’s Hospital of Shanghai University of Traditional Chinese Medicine between November 2022 and May 2023. The participants were randomly assigned to either a combination group (*n* = 35) or a sham control group (*n* = 35) using a random number table method. The combination group received warm acupuncture and Kegel exercise, whereas the sham control group underwent sham warm acupuncture and Kegel exercise. The study was approved by the Ethics Committee of the Seventh People’s Hospital of Shanghai University of Traditional Chinese Medicine (Approval No. 2022-7th-HIRB-048). All of the participants who attended the study signed a standard written consent form.

### Sample Size Calculation

**Fig. 1:**
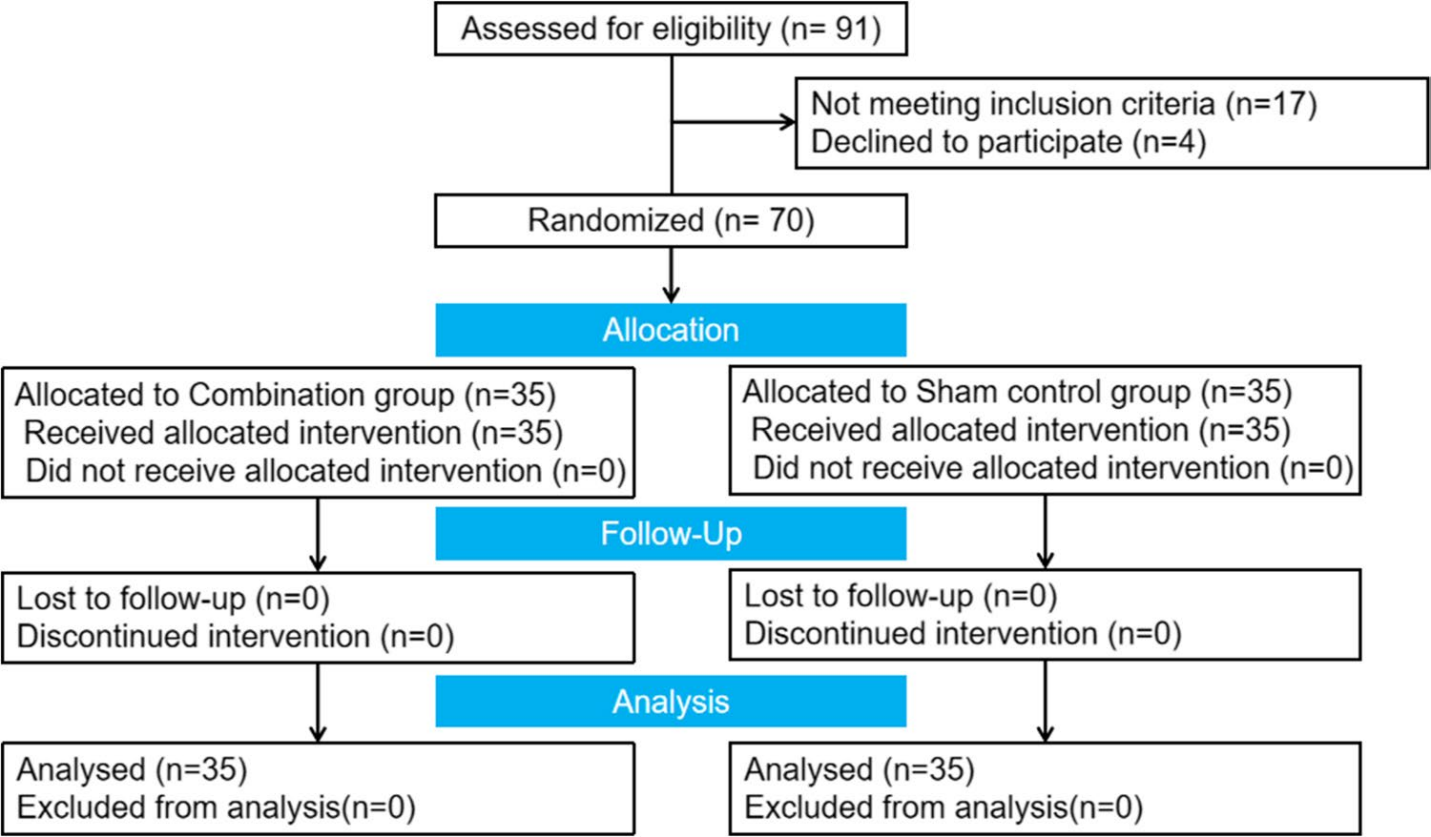
Participants’ flow diagram

According to the PFM strength, the main outcome indicator of this study, the Chi-squared test (goodness-of-fit tests: contingency tables) in the GPower3.1.9.7 software was used for estimation. The effect size was conservatively set at 0.3, the test level α = 0.05, the test power 1-β = 0.80, and the degree of freedom Df = 1. It is calculated that at least 88 subject samples are required. Considering a 10% loss to follow-up rate, at least 97 subjects are needed. However, owing to the impact of the COVID-19 epidemic during the study period, the sample size was insufficient, and only 70 patients were collected. Fortunately, pregnant women cherish medical resources more during special periods, and there are no cases of dropouts or loss of follow-up. The study design was controlled by the CONSORT checklist for RCTs, as depicted in Fig. 1.

### Diagnostic Criteria

#### Assessment of Incontinence Severity

To evaluate the severity of SUI symptoms, a 1-h pad test was administered in accordance with the guidelines established by the International Continence Society [15]. The results of the 1-h pad test indicate that mild SUI is characterized by leakage between 2 and 5 g, whereas moderate SUI is defined by leakage between 5 and 10 g.

##### Inclusion Criteria

1. Primiparous women who have undergone vaginal delivery with complete data
2. Forty-two days after delivery, the puerperas whose PFM strength of both class I and class II is less than grade 3
3. Age between 23 and 40 years old
4. Singleton full-term pregnancy
5. Body mass index between 18.5 and 23.9
6. Newborn birth weight between 2.5 and 4.0 kg [16]
7. No postpartum hemorrhage 2 h after delivery
8. Voluntary signing of the informed consent form

##### Exclusion Criteria Include

1. Severe SUI
2. A history of PPFD or other neuromuscular disorders before pregnancy
3. Failure to complete lochia within 42 days after delivery, severe primary diseases of the heart, liver, kidney, cerebrovascular system, or other systems, or gestational diabetes
4. Chronic cough
5. History of pelvic surgery
6. Severe needle phobia
7. Abnormal fetal position, cephalopelvic disproportion, or placental abruption during delivery
8. Episiotomy or other instrumental delivery, and third-degree or higher perineal laceration during delivery

#### Randomization Method

The SPSS25.0 random number generator was used to generate 70 random numbers within the range 0–1 (the number of decimal places is set to 2). Use the visual binning function to divide the random numbers into two groups, and obtain t 35 cases of the sham control group and the combination group. Seventy envelopes were taken, with numbers 1–70 written on the outside of the envelope, and a slip of paper inside. On the slip of paper, the corresponding group and the treatment method of this group are written according to the random number method. The pregnant women receive the envelopes with the number in the order of inclusion. The subjects, evaluators, and statistical analysts were not aware of the specific grouping situation, and owing to the nature of the intervention, acupuncturists could not be blinded.

### Intervention Methods

#### Kegel Exercise

The participants were directed to assume a supine position and engage in perineal and anal contractions for a duration of 3 s, followed by a relaxation period of equal length. The ratio of contraction and relaxation times is 1:1. Upon executing maximum force contractions for three consecutive instances, the participant was advised to completely relax for a duration of 6 s. This alternating cycle is to be repeated for a total of 10 min, thrice daily, over a period of 4 weeks. Both groups of subjects were asked to complete the same Kegel exercise plan.

In order to improve the quality and compliance of Kegel exercise, after the first acupuncture (sham acupuncture) treatment, the doctor explained the anatomical structure and function of the PFM to each mother in simple and easy-to-understand language, and put on disposable sterile plastic gloves. Two fingers were inserted into the subject’s vagina to provide feedback to the subject on PFM activity through finger palpation. The first Kegel exercise instruction takes 20 min or more, and after each acupuncture (sham acupuncture) treatment, the Kegel exercise instruction is given until the subject can master and control the PFM proficiently. All the above exercise guidance times are also included in the daily Kegel exercise plan for the subject. The rest of the time, the subject performed Kegel exercises at home. We provided them with written instructions and educational videos, and asked them to clock in the WeChat mini-program three times a day to improve their compliance with Kegel exercises.

#### Warm Acupuncture

**Fig. 2:**
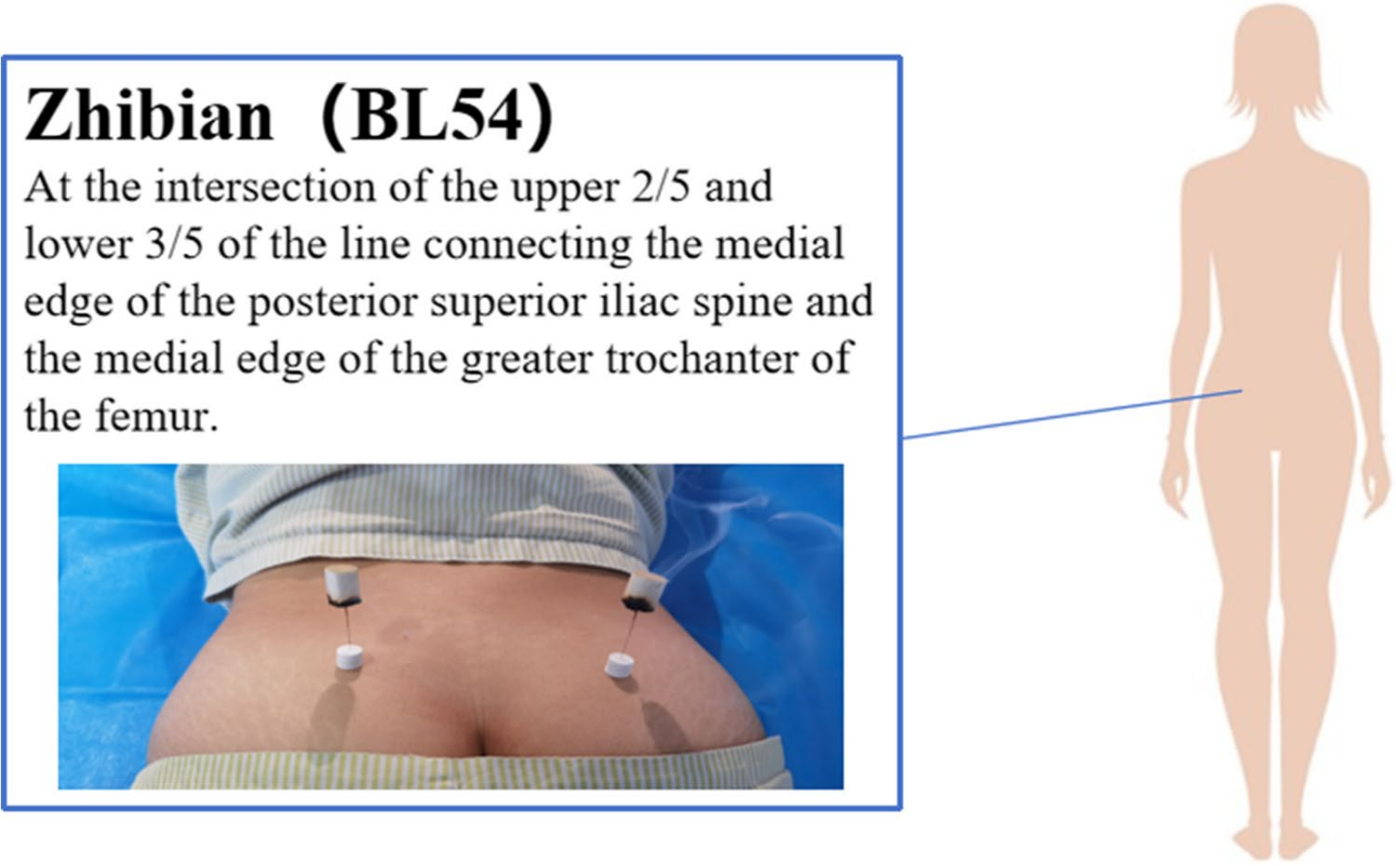
Zhibian acupoints

Bilateral Zhibian acupoints were selected. The Zhibian acupoint is located at the intersection of the upper two fifths and the lower three fifths of the line connecting the medial edge of the posterior superior iliac spine and the medial edge of the greater trochanter of the femur, as depicted in Fig. 2. The subjects assumed a prone position and underwent disinfection with 75% ethanol. A 0.45-mm × 100-mm needle was then inserted through the adhesive pad from the Zhibian (BL54) acupoint and directed straight down for approximately 70 mm. The resulting needle sensation, characterized by soreness, numbness, and distension, was expected to radiate toward the perineum or vulva. Subsequently, a small hole was created in the center of a 1.8-cm × 2.0-cm moxa stick using a toothpick, and the moxa stick was affixed to the needle handle and ignited for moxibustion. Following the completion of the moxa stick burning, the moxa ash was extracted, and the needle was retained for a duration of 30 min. In contrast, the individuals in the sham control group were positioned in a prone stance, sanitized with 75% ethanol, and subjected to the insertion of a 0.35-mm × 25-mm flat needle through the adhesive pad, which contacted the skin at the Zhibian acupoint. Although the parturient experienced discomfort, the needle tip did not penetrate the skin. The remaining steps were identical to those employed in the warm acupuncture procedure.

The treatment frequency is once every 2 days, three times a week, and the course of treatment is 4 weeks, a total of 12 times. Kegel exercise guidance and warm needle therapy were completed by two professional physicians respectively after standardized training. Both groups of subjects received their first treatment on the 43rd day postpartum and started learning Kegel exercise.

### Observational Indices

#### Main Outcome Indices: Assessment of PFM Strength Measurement

The measurement and evaluation of PFM strength are conducted through the evaluation method of the pelvic floor rehabilitation therapy instrument proposed by the “Expert Consensus on Diagnosis and Treatment of Vaginal Relaxation (2020 Edition),” production number: SH200192, specification: SOKO 900II, from Beijing Hailongma Technology Co., Ltd. Following bladder evacuation, the balloon is positioned at the bladder neck within the vagina, and the patient is directed to perform contractions and relaxation of the PFM. The strength of pelvic floor type I muscle fibers is assessed on a scale ranging from 0 to 5. A grade of 0 is assigned when the contraction surpasses the peak but does not endure for any length of time. A grade of 1 is given when the contraction lasts for 1 s, and subsequent grades are assigned based on increasing durations of contraction, with a duration of 5 s resulting in a grade 5. The strength of type II muscle fibers in the pelvic floor is assessed on a scale ranging from 0 to 5. A grade of 0 is assigned if the contraction does not surpass the peak, whereas a grade of I is given if it exceeds the peak once. The grading system continues in this manner, with a grade of 5 being awarded if the contraction exceeds the peak five times. In cases where the muscle strength falls below grade 3, it is advisable to undergo pelvic floor rehabilitation therapy.

Alterations in the strength of type I and type II muscle fibers were observed in two cohorts of postpartum women, both prior to and following treatment.

#### Secondary Outcome Indices

##### Pelvic Floor Ultrasound Examination

Parturients are instructed to empty their bladder before the examination. A routine scan is conducted with a color Doppler ultrasound diagnostic instrument (Affiniti70W model, PHILIPS company, Netherlands) set at a frequency range of 3.5–6.0 MHz to ensure the parturient’s steady respiration. The Urethral Rotation Angle (URA), Bladder Neck Descent (BND), and Retrovesical Angle (RVA) during Valsalva maneuver are quantified, wherein the parturient sustains breath and applies maximal abdominal pressure, both pre- and post-treatment. The data are collected three times and the mean value is computed.

##### Measurement of Pelvic Floor Ultrasound Parameters

**Fig. 3:**
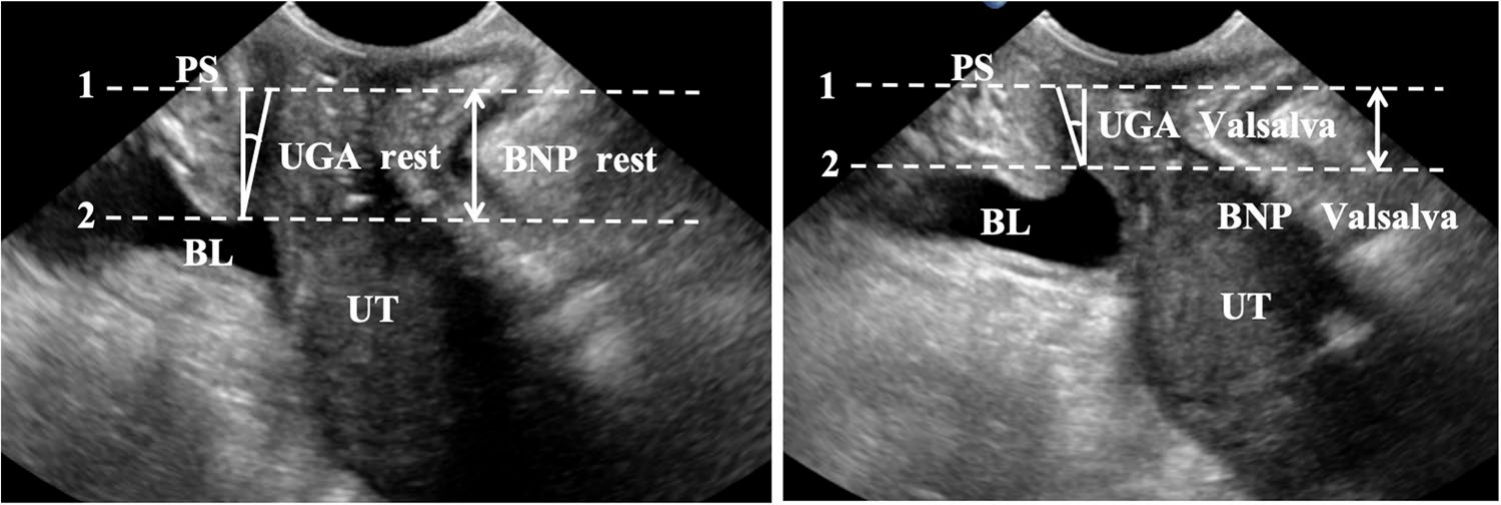
Measurement of the Urethral Rotation Angle (*URA*) and Bladder Neck Descent (BND). The left side illustrates the Urethral Gradient Angle (UGA) and the Bladder Neck Position (BNP) at rest, and the right side illustrates the UGA and the BNP during the Valsalva maneuver. The URA is the summation of the two angles. The horizontal line of the lower edge of the pubic symphysis is represented by the *dotted line 1*; *dotted line 2* is the horizontal line of the bladder neck position. *PS* pubic symphysis, *BL* bladder, *UT* uterus, *UGA* Urethral Gradient Angle, *BNP* bladder neck position

**Fig. 4:**
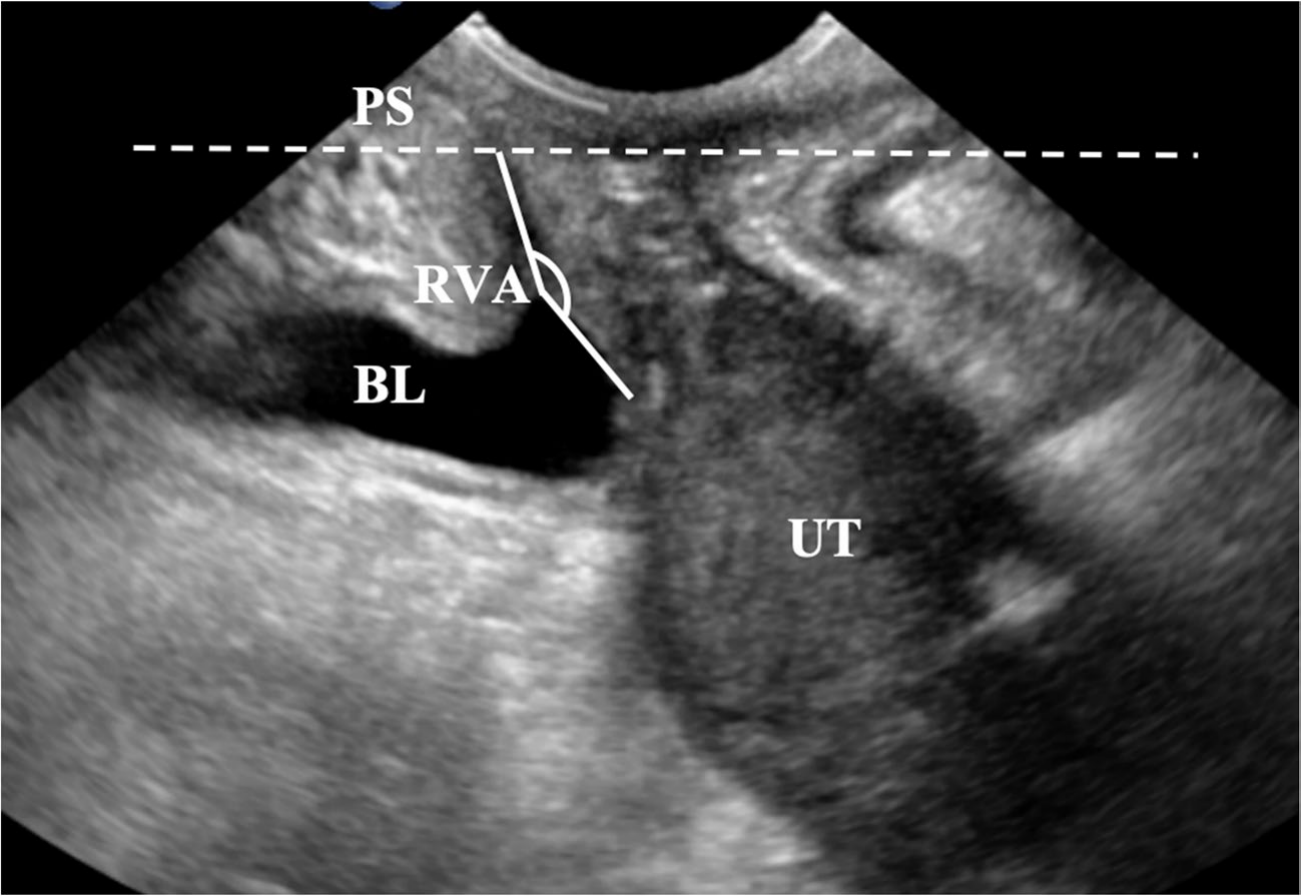
Measurement of the Retrovesical Angle during Valsalva maneuver. The *dotted line* is the horizontal line of the lower edge of the pubic symphysis. *PS* pubic symphysis, *BL* bladder; *UT* uterus, *RVA* Retrovesical Angle

Studies have shown that three pelvic floor anatomical parameters, URA, BND and RVA, are significantly related to the mode of delivery or the incidence of postpartum urinary incontinence [17, 18]. The URA refers to the angle of the urethra at rest and during Valsalva maneuver, as illustrated in Fig. 3. BND is defined as the difference between the position of the bladder neck during maximum Valsalva maneuver and at rest, as depicted in Fig. 3. The RVA denotes the angle between the proximal urethra and the posterior wall of the bladder trigone, as shown in Fig. 4. According to the Green classification of ultrasonic cystocele: URA > 45°, RVA > 140° are the pelvic floor ultrasound diagnostic criteria for cystocele [19]. BND "has no specific normal definition" although a boundary between 15–25 mm has been proposed to define BND [20, 21].

##### Assessment of Pelvic Floor Function

The assessment of pelvic floor function in the two groups of postpartum women before and after treatment was conducted using the International Consultation on Incontinence Questionnaire-Short Form (ICIQ-SF) and the Pelvic Floor Dysfunction Inventory-20 (PFDI-20).

The PFDI-20 [22] scale evaluates the condition of the lower gastrointestinal tract (eight questions), lower genital tract (six questions), and lower urinary tract (six questions) of the subjects in the past 3 months, totaling 20 questions. The degree is indicated by numbers (0 point for no symptoms, 1 points for symptoms without effects, 2 points for mild effects, 3 points for moderate effects, and 4 points for severe effects). The total score ranges from 0 to 300. Higher scores indicating more severe symptoms. Among them, asymptomatic (0 points), mild (1–15 points), moderate (16–34 points), and severe (35–40 points).

The ICIQ-SF [23] consists of four questions. The first three questions are scored from the frequency, degree, and impact on daily life of puerpera. The fourth question is the investigation of the occurrence of urinary incontinence. The total score ranges from 0 to 21. The higher the score, the more severe the symptoms. ICIQ-SF score of ≥ 6 points is defined as symptomatic urinary incontinence, and a score < 6 points is defined as asymptomatic urinary incontinence.

##### Assessment Criteria of Cumulative Effect

According to similar studies by previous researchers, after treatment, if the PFM strength is ≥ level 3, it is effective, and if it is still < level 3, it is invalid [13, 14].

The efficacy of urinary incontinence treatment was evaluated through a comparative analysis of the 1-h urine pad test for leakage before and after treatment in two groups of pregnant women [24]. The definition of cure is the absence of SUI and subjective feeling of no leakage. Effective treatment is defined as a significant reduction of more than 50% in the 1-h urine pad test leakage volume compared with before. Conversely, ineffective treatment is defined as a reduction of less than 50% in the 1-h urine pad test leakage volume compared with before. The total effectiveness rate is calculated as the sum of the cure rate and the effective rate.

### Statistical Analysis

The data analysis was executed through the utilization of SPSS 25.0 software. The measurement data were presented as the mean value accompanied by the standard deviation (SD), and the comparisons between groups were conducted by means of an independent samples *t* test. The count data were expressed as rates or proportions, and the comparisons between groups were conducted using a Chi-squared test. A statistical significance level of *p* < 0.05 was deemed appropriate.

## Results

### Comparison of General Clinical Data

The general clinical data, including age, gestational week, BMI before pregnancy, and newborn weight, did not exhibit a statistically significant difference between the two groups (*p* > 0.05), indicating comparability of the data (Table 1).

**Table 1:** Comparison of general clinical data between the two groups

Variables	Combination group (*n* = 35)	Sham control group (*n* = 35)	*p* value
Age, years	28.86 (3.46)	27.89 (3.46)	0.215
Gestational week, weeks	38.51 (1.92)	38.63 (1.24)	0.768
BMI before pregnancy, kg/m^2^	21.23 (1.61)	21.01 (1.73)	0.584
Newborn weight, g	3,234.57 (329.42)	3,238.00 (334.51)	0.966

Data are presented as mean (SD)

### Changes of PFM Strength

Prior to intervention, no statistically significant differences in PFM strength were observed between the two groups (*p* > 0.05). Following a 4-week intervention, the combination group exhibited a recovery rate of 88.57% (31 out of 35) for type I PFM strength, compared with 65.71% (23 out of 35) in the sham control group. The combination group demonstrated a superior effect compared with the sham control group (*p* < 0.05). Additionally, the combination group exhibited a recovery rate of 91.43% (32 out of 35) and 68.57% (24 out of 35) for type II PFM strength, which was significantly better than the control group (*p* < 0.05).

### Comparison of the URA, BND, and RVA during the Valsalva Maneuver

Prior to intervention, there existed no statistically significant distinction in URA, BND, and RVA during Valsalva maneuver between the two groups (*p* > 0.05). Following intervention, the combination group exhibited lower URA, BND, and RVA during Valsalva maneuver in comparison with the control group (*p* < 0.05; Table 2). These findings suggest that the recovery of the pelvic floor in the combination group is superior to that of the control group.

**Table 2:** Analytical statistics before/after intervention between two groups

Variables		Combination group (*n*=35)	Sham control group (*n*=35)	*P* value
Urethral Rotation Angle, °	Before intervention	35.58 (14.35)	33.63 (13.24)	0.555
	After intervention	22.86 (11.39)	28.28 (10.30)	0.041**
	*P* value	0.000*	0.000*	
Retrovesical Angle during Valsalva maneuver, °	Before intervention	154.39 (16.90)	156.24 (13.56)	0.614
	After intervention	135.60 (12.82)	143.69 (13.75)	0.013**
	*P* value	0.000*	0.000*	
Bladder Neck Descen, mm	Before intervention	8.00 (4.04)	8.46 (4.54)	0.658
	After intervention	4.77 (4.44)	6.86 (3.39)	0.031**
	*P* value	0.000*	0.034*	
ICIQ-SF scores	Before intervention	5.41 (4.37)	4.77 (3.60)	0.508
	After intervention	2.09 (2.59)	3.82 (3.26)	0.016**
	*P* value	0.000*	0.039*	
PFDI-20 scores	Before intervention	21.34 (17.26)	19.13 (15.45)	0.573
	After intervention	6.25 (8.60)	11.24 (11.80)	0.047**
	*P* value	0.000*	0.000*	

Data are presented as mean (SD)

*ICIQ-SF*, International Consultation on Incontinence Questionnaire-Short Form; *PFDI-20*, Pelvic Floor Dysfunction Inventory-20

*P < 0.05, comparison before and after intervention in this group

**P < 0.05, comparison between combination group and sham control group after intervention

### Changes in Quality of Life

Prior to intervention, there were no discernible disparities in the ICIQ-SF and PFDI-20 scores between the two groups (*p* > 0.05). Following a 4-week intervention, both groups exhibited lower ICIQ-SF and PFDI-20 scores in comparison with their pre-intervention scores (*p* < 0.05). Specifically, the combination group demonstrated significantly lower ICIQ-SF and PFDI-20 scores than the sham control group after the 4-week intervention (*p* < 0.05), as presented in Table 2.

### Comparison of Urinary Incontinence

Among the 70 subjects collected in this study, 32 subjects in the combination group had SUI, and 31 subjects in the control group had SUI. The remaining 7 women reported that they had no pelvic floor discomfort or urinary leakage. The combination group exhibited an effective rate of urinary incontinence of 93.75% (31 out of 32), whereas the control group had a rate of 80.65% (24 out of 31). The results indicate that the curative effect of the combination group was superior to that of the control group (*p* < 0.05).

## Discussion

Women who have experienced pregnancy and childbirth are at an increased risk of pelvic floor dysfunction [25]. According to research, the act of pulling and compressing the fetal head during vaginal delivery has been found to result in nerve damage in the perineum, as well as an elevated risk of injury to the levator ani muscle and increased mobility of the bladder neck [26]. Although Kegel exercise is a commonly used clinical postpartum pelvic floor rehabilitation method [27], the quality of completion of Kegel exercises by postpartum women is influenced by various factors, and participants in this study expressed fatigue and burden after performing 30 min of Kegel exercises daily; more importantly, incorrect training can even increase abdominal pressure, leading to muscle pain [28, 29]. Conversely, warm acupuncture therapy was deemed more tolerable owing to its heightened comfort level, gentle stimulation, and lack of impact on breastfeeding, rendering it a more favorable option.

The potential mechanisms underlying the effects of warm acupuncture on PPFD may be associated with the inferior location of the perineal nerves in relation to the Zhibian acupoint. Research has demonstrated that acupuncture stimulation of these nerves can augment the contraction of PFMs, resulting in enhanced urinary control and strengthened pelvic floor musculature [30]. Furthermore, the perineal nerves are a subdivision of the pelvic plexus nerves, and the sensation elicited by needle insertion may propagate through the perineal nerves to the pelvic plexus nerves, subsequently providing feedback to the primary urinary control center located in the lumbosacral spinal cord or even higher to the cerebral cortical urinary control center, thereby exerting a favorable regulatory influence on the urination center [31].

In the process of warm acupuncture, thermal energy is consistently conveyed into the body through both the needle shaft and the needle body. The amalgamation of mechanical stimulation from acupuncture and thermal stimulation from moxibustion on the needle body results in a compound stimulation, which generates a novel effect. This concentrated heat enables deeper penetration into the body’s layers, thereby producing thermal stimulation on deep tissues and ultimately restoring pelvic floor function [32, 33]. According to a recent study [34], warm acupuncture has been found to enhance the conductivity of meridians, leading to increased electrical currents and reduced impedance in the meridians. That is to say, compared with ordinary acupuncture, warm acupuncture can cause more severe soreness, numbness, and swelling, and enhance the therapeutic effect of acupuncture. Traditional Chinese Medicine posits that warm needle acupuncture, a combination of acupuncture and moxibustion, can effectively restore weakened bodily functions, offering gentle stimulation, no toxic side effects, and greater comfort for postpartum women.

Before treatment, the average value of the RVA during Valsalva maneuver in both groups was greater than 140°, and the combination group decreased to below 140° after treatment, which was significantly smaller than that in the sham control group (*p* < 0.05). Studies by Wen et al. showed enlarged RVA with more extension and less rotation of the bladder neck and proximal urethra [35]; our research results show that the URA in both groups was smaller than before treatment, which is contrary to the research results of Wen et al. [35]. However, in this study, the efficacy of maternal urinary incontinence was significantly improved. It is considered that our treatment may improve PFM strength and enhance the tightening effect on the urethra. Although the average URA of the two groups of subjects before treatment did not exceed 45° and the BND did not exceed 15 mm, our study can only prove that warm acupuncture combined with Kegel exercise can reduce the URA and BND of primiparous women with vaginal delivery; it does not mean that this treatment method can make them appear clinically improved corresponding to the diagnostic criteria of pelvic floor ultrasound. But this is only the first step in research on the promotion of female pelvic floor function recovery by warm acupuncture combined with Kegel exercise. On the one hand, all the puerperas we treated were primiparous, younger, and had no serious injuries during delivery. On the other hand, our puerperas only had decreased PFM strength and did not have severe pelvic floor dysfunction. Therefore, The URA and BND of the parturients did not reach the thresholds corresponding to the diagnostic criteria of pelvic floor ultrasound. We will continue to study the efficacy of this treatment method for the treatment of postpartum severe pelvic floor dysfunction.

The standard deviation of the ICIQ-SF questionnaire was 3.98, and the minimally important difference (MID) was estimated to be 2 based on the standard deviation; the standard deviation of the PFDI-20 questionnaire was 16.30, and the MID was estimated to be 8 based on the standard deviation. After the intervention, the proportion of pregnant women with PFDI-20 questionnaire scores less than MID in the combination group was 62.5% (21 out of 35), which was more than 46.57% (15 out of 35) in the sham control group, but the difference between the two groups was *p* = 0.232, which was not statistically significant. The ICIQ-SF questionnaire had the same results: in the combination group less than the MID accounted for 56.25% (18 out of 35), more than the sham control group (34.37%; 11 out of 35), but the difference was still not statistically significant (*p* = 0.145). Therefore, from the perspective of statistical analysis, it cannot be considered that parturients felt that their pelvic floor function was recovering. However, judging from the evaluation content of the PFDI-20 and ICIQ-SF scales, the clinical manifestations related to pelvic floor dysfunction of the two groups of puerperas did indeed decrease. According to communication between doctors and puerperas, the puerperas did feel that they were getting better. And if calculated according to the standard of PFDI-20 < 15 points and ICIQ-SF < 6 points, the ICIQ-SF questionnaire scores of the combination group with less than 6 points accounted for 97.1% (34 out of 35) more than the sham control group (68.6%; 24 out of 35), *p* = 0.003 (the difference was statistically significant, and it can be considered that the clinical efficacy of the combination group was better than that of the sham control group.

At present, most acupuncture treatments for PPFD use a combination of multiple acupoints [36, 37], and it is difficult to distinguish the effectiveness of individual acupuncture points. The puerpera is already weak after childbirth. The theory of traditional Chinese medicine is that too many selected acupoints may increase the burden on the body. Therefore, this study first introduced the theory of single-point therapy. Although there have been studies showing that Zhinbian point acupuncture can promote the improvement of pelvic floor function, when we designed the study, we were still worried that the efficacy might not be good; therefore, we excluded women with third-degree perineal lacerations and episiotomy. This is also one of the limitations of this study. Therefore, the therapeutic effect of a single point on severe postpartum pelvic floor injuries is still uncertain and needs further exploration.

In addition, it should be ensured that the damage to the pelvic floor of the two groups of subjects due to childbirth is at the same baseline level as far as possible, in order to improve the accuracy and scientificity of the research results. The inclusion and exclusion criteria of this study controlled for the pre-pregnancy BMI and newborn body weight within a very strict range, and mothers who did not fall within this limit were excluded. This was an ill-considered aspect of our study design. This also provides new ideas for future research. It may be a new research direction to analyze the impact of pre-pregnancy BMI and newborn body weight on the efficacy of acupuncture in promoting postpartum pelvic floor function recovery.

Postpartum pain is also an indicator that is frequently observed clinically for postpartum women. At the start of the study, the VAS score was an indicator of postpartum pelvic pain, but this may be because this study placed strict restrictions on the newborn’s body weight and the degree of postpartum perineal tearing; not all the women had serious pelvic floor injuries during delivery. Among the 70 women, only 3 had postpartum pubic pain. Statistical analysis was not possible and therefore this was not analyzed.

## Conclusions

In conclusion, the combination of warm acupuncture and Kegel excises has been found to be effective in enhancing PFM strength, restoring pelvic floor anatomy, and improving pelvic floor function among postpartum women. This study provides evidence for the efficacy of a single acupoint, thereby contributing to the precision and standardization of acupuncture.

## Data Availability

The datasets used and/or analyzed during the current study are available from the corresponding author on reasonable request.
